# The Impact of Routine Transvaginal Ultrasound Measurement of the Cervical Length on the Prediction of Preterm Birth: A Retrospective Study in a Tertiary Hospital

**DOI:** 10.1055/s-0041-1726053

**Published:** 2021-05-12

**Authors:** Joana Patricia Rodrigues Félix Peixoto de Almeida, Carla Maria Magno Bartosch, Alexandra Matias Pereira Cunha Coelho Macedo

**Affiliations:** 1 Department of Gynecology and Obstetrics, Faculdade de Medicina, Hospital Pedro Hispano, Universidade do Porto, Porto, Portugal; 2 Department of Pathology, Instituto Português de Oncologia do Porto, Porto, Portugal; 3 Department of Gynecology and Obstetrics, Faculdade de Medicina, Centro Hospitalar Universitário São, Universidade do Porto, Porto, Portugal

**Keywords:** preterm birth, preterm birth screening, transvaginal ultrasound cervical length, cervical length cut-off, parto pré-termo, rastreio parto pré-termo, comprimento cervical por ecografia transvaginal, valor de referência do comprimento cervical

## Abstract

Preterm birth (PTB) is a major obstetric problem associated with high rates of neonatal morbidity and mortality. The prevalence of PTB has not changed in the last decade; thus, the establishment of a screening test and effective treatment are warranted. Transvaginal ultrasound measurement of the cervical length (TUCL) has been proposed as an effective method to screen pregnant women at a higher risk of experiencing PTB.

**Objective** To evaluate the applicability and usefulness of second-trimester TUCL to predict PTB in a cohort of Portuguese pregnant women.

**Methods** Retrospective cross-sectional cohort study including all singleton pregnant women who performed their second-trimester ultrasound (between weeks 18 and 22 + 6 days) from January 2013 to October 2017 at Centro Hospitalar Universitário São João.

**Results** Our cohort included 4,481 women. The prevalence of spontaneous PTB was of 4.0%, with 0.7% occurring before the 34th week of gestation. The mean TUCL was of 33.8 mm, and percentiles 3, 5 and 10 corresponded to TUCLs of 25.0 mm, 27.0 mm and 29.0 mm respectively. The multiple logistic regression analysis, including maternal age, previous PTB and cervical surgery showed a significant negative association between TUCL and PTB, with an odds ratio (OR) of 0.92 (95% confidence interval [95%CI]: 0.90–0.95; *p* < 0.001). The use of a TUCL of 20 mm is the best cut-off, when compared with the 25-mm cut-off, improving the prediction of risk.

**Conclusion** The present study showed an inverse association between TUCL and PTB, and that the inclusion of other risk factors like maternal age, previous PTB and cervical surgery can improve the screening algorithm. Furthermore, it emphasizes that the TUCL cut-off that defines short cervix can differ according to the population.

## Resumo

O parto pré-termo (PPT) é uma grande complicação obstétrica que se associa a elevadas taxas de morbimortalidade neonatal. A sua prevalência não tem alterado na última década, sendo esencial determinar uma forma de rastreio e tratamento eficaz. A medição ecográfica transvaginal do comprimento cervical tem sido proposta como um método eficaz de rastreio das grávidas com risco aumentado de PPT.

**Objetivo** Avaliar a aplicabilidade e utilidade da medição ecográfica transvaginal do comprimento cervical na previsão de PPT numa amostra de grávidas portuguesas.

**Método** Estudo de coorte retrospectivo incluindo todas as grávidas com gestação unifetal que realizaram ecografia do 2° trimestre (de 18 a 22semanas + 6 dias) no Centro Hospitalar Universitário de São João entre janeiro de 2013 e outubro de 2017.

**Resultados** A nossa amostra incluiu 4.481 mulheres. A prevalência de PPT espontâneo foi de 4,0%, sendo que 0,7% ocorreu antes das 34 semanas de gestação. A média do comprimento cervical por ecografia transvaginal foi 33,8 mm, e os percentis 3, 5 e 10 da amostra corresponderam a comprimentos cervicais de 25,0 mm, 27,0 mm e 29,0 mm, respetivamente. A regressão logística múltipla, que incluiu a idade materna, PPT anterior e antecedentes de conização, demonstrou uma associação estatisticamente significativa entre o comprimento cervical e o risco de PPT, com um risco relativo de 0,92 (intervalo de confiança de 95% [IC95%]: 0.90–0.95; *p* < 0.001). A utilização de um valor de referência de comprimento cervical de 20 mm, quando comparado com o valor de referência de 25 mm, melhora a previsão do risco de PPT.

**Conclusão** Este estudo demostra uma associação entre o comprimento cervical avaliado por ecografia tranasvaginal e o risco de PPT, e salienta que a inclusão de outros fatores de risco, como idade materna, PPT anterior e antecedentes de conização podem melhorar o algoritmo de rastreio. Realça ainda que o valor de comprimento cervical utilizado para definir “colo curto” varia de acordo com a população em estudo.

## Introduction

The World Health Organization (WHO) defines preterm birth (PTB) as a delivery that occurs before the 37th week of gestation. It can occur spontaneously or due to medical induction (iatrogenic). Poorly understood to date, spontaneous PTB is a heterogeneous syndrome with multiple underlying pathophysiologic events and causes,.

Approximately 11% of infants worldwide are born too soon, corresponding to 15 million premature newborns every year. 1 2 3 The prevalence ranges from 5% in European developed countries to 18% in certain African countries, but these international differences may reflect variations in definitions rather than a true epidemiological difference. For example, the method to determine the gestational age and different viability limits can influence this rate. In Portugal, the prevalence of singleton live preterm newborns is of 7.4%. 4

Despite all advances in medicine, PTB is still an important health problem, and the leading cause of neonatal mortality. Prematurity is associated with multiple neonatal complications and long-term morbidity. 5 6

Fetal development is a continuum, and the risk of perinatal complications is inversely related to the gestational age at delivery. For this reason, some experts recommend a subclassification of PTB into early PTB (< 34 weeks) and late PTB (between 34 weeks and 36 weeks + 6 days), as the negative impact is different in the two groups. 7 Infants born before the 32th week of gestation represent less than 2% of all premature births, but they contribute to 50% of the overall perinatal mortality. 8

Preterm birth is such a major economic and social burden that its reduction is one of the Millennium Development Goals established by the United Nations. 9 Unfortunately, despite all efforts, the rate of prematurity has not changed in the past 30 years, and, in 2016, the WHO included PTB as one of the top-10 priority research areas. 10 11

During the last years, many risk scores have been proposed to predict PTB, but they all have a low sensitivity and poor positive predictive value (PPV). 12 13 The history of previous spontaneous PTB, for example, is the most significant risk factor known, but only 10% to 15% of PTBs occur after a previous event. 14 15

As the majority of spontaneous PTBs occur in low-risk pregnancies, Andersen et al. 16 (1990) proposed the use of transvaginal ultrasound measurement of the cervical length (TUCL) as a predictor of PTB. Since then, the technique has been well standardized, and its reproducibility, confirmed. 17 18

The risk of experiencing PTB is inversely correlated to the cervical length, but the ideal cut-off for clinical use is still controversial. 16 19 20 21 22 23 By definition, a cervical length below the 10th centile for gestational age is considered “short.” This value varies according to the gestational age, the populational distribution of TUCL, and the prevalence of PTB. In the initial trials, the 10th centile was of 25 mm; therefore, this cut-off has been widely used. 15 24 25 26 27 Since then, many cut-offs (from 15 mm to 30 mm) have been proposed, but none is consensual.

The main objective of the present study was to evaluate the applicability and usefulness of second-trimester TUCL to predict PTB in Portuguese pregnant women. We analyzed the distribution of TUCL in our cohort and determined the prevalence of short cervix using different cut-offs. Furthermore, we developed models to estimate the best TUCL cut-off in our cohort and improve its usefulness.

## Methods

The present was an observational, retrospective cross-sectional cohort study carried out at the Obstetrics and Gynecology Department of Centro Hospitalar Universitário São João (CHUSJ), Portugal, after approval by the hospital's ethics committee (CES 81-17).

We included all singleton pregnant women who underwent the second-trimester ultrasound (the 18th week to the 22nd week + 6 days, determined by the crown-rump length before the 14th week) from January 2013 to October 2017 in this hospital. Delivery in the same institution and the existence of delivery data were also inclusion criteria. We excluded all women that had induced PTBs for medical reasons (including premature rupture of membranes), cervical cerclage performed prior to screening, diagnosis of chorioamnionitis, and deliveries before the 24th week.

**Fig. 1:**
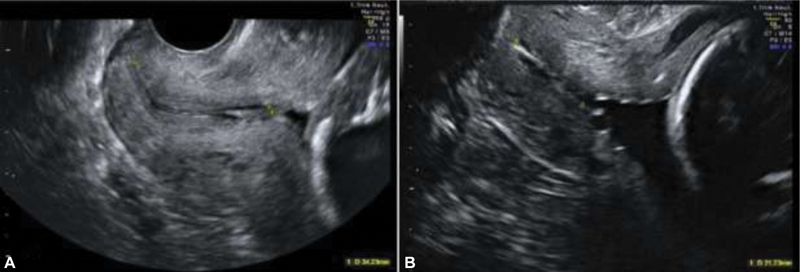
Transvaginal ultrasound measurement of the cervical length (TUCL). ( **A** ) Normal cervix; ( **B** ) short cervix.

The ultrasound exams at CHSJ are performed by obstetricians with accreditation from the Fetal Medicine Foundation (FMF) for cervical assessment. However, because the universal screening of TUCL is not mandatory, all ultrasound images available through the Astraia software (Astraia Software, GmbH, Munich, Germany) were reviewed in order to identify patients with an ultrasound image that complied with standard the rules of the FMF, which recommends the use of a transvaginal probe with the identification of the sagittal view of the cervix, occupying 75% of the image. Identification of the internal os, external os and cervical canal is essential. The measurement is performed in a straight line between the external and internal os. Care should be taken to distinguish between the cervical canal and the lower uterine segment ( Fig. 1 ).

At our hospital, all pregnant women with TUCLs ≤ 25 mm are considered to have a short cervix, and vaginal progesterone or the Arabin (Dr. Arabin GmbH & Co., Witten, Germany) pessary is suggested.

Maternal characteristics, medical history, obstetric history and delivery data were obtained from the database of the Obstetrics and Gynecology Department through the Obscare software. This data was compiled using the Statistical Package for the Social Sciences (SPSS, IBM Corp., Armonk, NY, US) software, version 24, for the statistical analyses. The continuous variables were expressed as means ±  standard deviations (SDs), and frequencies and percentages were used to describe the categorical variables.

The frequencies of PTB were calculated according to different groups of cervical length measurements. The diagnostic ability of different TUCL cut-offs was evaluated in terms of sensitivity, specificity, PPV, negative predictive value (NPV), and the area under the curve (AUC).

An exploratory univariate analysis of clinical and demographic global data was first performed to determine the variables that predicted PTB and those associated with TUCL. All of the hypothesis tests conducted were two-tailed, and they included the Student *t* -test, the Chi-squared (χ ^2^ ) test, and the Fisher exact test, as appropriate. Then, we developed a multivariate logistic regression model aiming to predict PTB as the outcome, using the TUCL as the explanatory variable, and including the main effects of maternal age, previous PTB and cervical surgery. For all of these analyses, values of *p* < 0.05 were considered statistically significant.

To select an optimal TUCL cut-off, we used the maximum likelihood and a confidence interval based on a likelihood ratio test. The likelihoods were calculated for a series of our multivariate logistic regression model using all TUCL cuto-ffs between 8 mm and 50 mm. The 95% lower and upper confidence bounds were determined as parameter values that reduce the maximum likelihood by χ ^2^ (0.05,1)/2 = 1.92. Using this optimal TUCL cut-off, we then assessed the potential differential effects across subgroups of risk factors using a stratified analysis. The effect modification among strata was checked using a test of homogeneity. Adjusted estimates were calculated using the Cochran-Mantel-Haenszel method.

In Portugal, the most used cut-off is TUCL ≤ 25 mm. In order to compare our optimal cut-off with the 25-mm cut-off, we classified each woman into groups of predicted probabilities derived from corresponding multivariate models using each cut-off. We then cross-classified these groups and compared them to the observed proportions of events in each group.

## Results

During the aforementioned period, 8,016 women underwent a routine second-trimester ultrasound and delivery at CHUSJ. In total, 3,476 women were excluded from this group for the following reasons: delivery before the 24th week (n = 5),; medically-induced PTB (n = 241); cervical cerclage prior to ultrasound (n = 19); diagnosis of chorioamnionitiss (n = 3); absence of cervical length measurement (n = 958); and images of the cervical length measurement that did not comply with FMF recommendations (n = 2,426). Within the latter group, the major reason for exclusion was a transabdominal measurement (n = 1,275), instead of the transvaginal approach preconized by the FMF. Some women presented more than one exclusion criteria.

Our final cohort consisted of 4,481 women with a mean age of 30.7 ±  5.5 years. Primigravidae represented 45.4% of the sample, and 56.2% had no previous delivery. Most of them had no medical (86.5%) or obstetric (96.7%) relevant background. Only 1.6% of these women had a previous spontaneous PTB, and 0.9% had history of cervical surgery, the 2 major known risk factors for PTB.

Spontaneous delivery occurred in 64.3% of the cases, and 76.4% of the women underwent vaginal delivery. The prevalence of spontaneous PTB prevalence of the original cohort (8,016 women) was of 6.9% (553), and, after applying the exclusion criteria (with the sample reduced to 4,481 women) the prevalence dropped to 4.0% (n = 179), mainly due to the exclusion of medically-induced PTB. In total, 96.0% (n = 149) of the cases of PTB occurred between the 34th and 37th weeks, and 0.7% (n = 30) occurred before 34th week of gestation. The maternal and clinical characteristics of our cohort are described in table 1 .

**Table 1:** Maternal and clinical characteristics of the study population

Maternal features	Medical background	Risk factors for Preterm birth
Age (years) Mean ± standard deviation: 30.7 ± 5.5 Min: 14 Max: 50 Body mass index (kg/m ^2^ ) Mean ± standard deviation: 24.6 ± 4.9 Obesity (> 30): 8.0% Years of schooling < 4th grade: 0.7% 4th to 12 ^th^ grades: 64.6% > 12 ^th^ grade: 34.7% Smokers: 12.9% Alcohol/drug users: 0.2%	None: 86.5% Uterine malformations: 0.3% Psychiatric disorders: 1.4% Sexually-transmitted diseases: 1.0% Cardiac or renal disorders: 1.0% Diabetes: 0.7% Hypertension: 2.6% Hypothyroidism: 5.2% Neoplasia: 0.8%	Spontaneous preterm birth: 1.6% Cervical surgery: 0.9% Short cervical length (≤25 mm) -» On 2 ^nd^ -trismester ultrasound: 3.0% Obstetric background None: 96.7% Preeclampsia: 0.9% Fetal death: 0.6% Fetal malformation: 0.4% 2 ^nd^ T abortion: 0.1%
**Obstetric intercurrences**	**Actual obstetric data**	**Time of delivery**
Fetal growth restriction: 3.9%	Primigravida: 45.4%	Mean: 39.2 weeks
Fetal malformation: 1.6%	Nullipara: 56.2%	Minimum: 24.2 weeks
Urinary infection: 3.2%	Assisted reproduction: 2.7%	Maximum: 42 weeks
Other infections: 4.4%	Labor induction: 35.7%	Term delivery (≥37 week): 96.0%
Hypertensive syndrome: 3.2%	Vaginal delivery: 76.4%	Preterm delivery (< 37 week): 4.0%
Gestational diabetes: 9.6%	Male newborn: 50.6%	Early preterm birth (< 34 ^th^ week): 0.7%
Surgery on 1 ^st^ /2 ^nd^ trimesters: 0.2%		Late preterm birth (≥ 34 ^th^ week): 3.3%

The mean gestational age at the time of the ultrasound was 21 weeks + 3 days, with a distribution of 0.2% (8) at 18 weeks, 0.5% (22) at 19 weeks, 12.4% (555) at 20 weeks, 66.5% (2979) at 21 weeks, and 20.5% (917) at 22 weeks. The mean TUCL was of 33.8 mm ±  4.8 mm (range: 3.0 mm to 53.0 mm). Percentiles 3, 5 and 10 corresponded to TUCLs of 25.0 mm, 27.0 mm and 29.0 mm respectively. Table 2 presents the frequency of term and preterm births across different TUCL intervals. Among pregnant women with PTB, the TUCL was significantly lower (mean: 31.6 mm; 95%CI: 30.7–32.5 mm) compared to the measurements of those with term birth (mean: 33.9 mm; 95%CI: 33.8–34.0 mm; *p* < 0.001).

**Table 2:** Distribution of preterm and term births across different cervical length intervals

Cervical length	Early preterm birth (< 34 weeks): n (%)	Preterm birth (< 37 weeks): n (%)	Term birth (≥37weeks): n (%)	Total: n (%)
< 15 mm	4 (13.3)	5 (2.8)	11 (0.3)	16 (0.4)
15.1 to 20 mm	3 (10.0)	4 (2.2)	11 (0.3)	15 (0.3)
20.1 to 25 mm	0 (0)	6 (3.4)	74 (1.7)	80 (1.8)
25.1 to 30 mm	3 (10.0)	30 (16.8)	506 (11.8)	536 (12.0)
≥ 30 mm	20 (66.7)	134 (74.9)	3,700 (96.5)	3,834 (85.6)

Even though the TUCL alone showed a high specificity to predict PTB, its diagnostic ability was limited by a very low sensitivity, with an AUC close to 0.5 for all different cut-offs studied, as depicted in table 3 .

**Table 3:** Sensitivity and specificity of the cervical length measurement to predict preterm birth and cumulative incidence of the different cut-offs

Cut-off	Sensitivity	Specificity	Area under the curve	Positive predictive value	Negative predictive value
15 mm	2.8	99.7	0.51	10.9	0.97
20 mm	6.2	99.5	0.53	11.5	0.94
25 mm	10.6	97.3	0.54	3.9	0.91
30 mm	30.7	80.1	0.55	1.5	0.86

The univariate analysis ( Tables 4 and 5 ) showed that maternal age ≥ 40 years, history of PTB, and cervical surgery were the main significant predictors of PTB. Additionally, history of PTB and previous cervical surgery were also associated with shorter TUCL, thus acting as confounders.

**Table 4:** Demographics comparing preterm and term births

		Preterm birth – n: 179 (4.0%)	Term birth – n: 4302 (96.0%)	*p* -value
Maternal age	Mean ± standard deviation	31.4 ± 6.0	30.7 ± 5.5	0.098 ^a^
	< 40 years old	161 (89.9%)	4122 (95.8%)	< 0.001 ^b^
	≥40 years old	18 (10.1%)	180 (4.2%)	
Body mass index	Mean (kg/m ^2^ )	2397 ± 5.3	24.6 ± 4.9	0.055 ^a^
Schooling	≤ 12th grade	111 (62.4%)	2807 (65.4%)	0.404 ^b^
	> 12th grade	67 (37.6%)	1485 (34.6%)	
Addictions	Smoking	21 (11.7%)	556 (12.9%)	0.640 ^c^
	Drugs	0 (0%)	4 (0.1%)	1.000 ^c^
	Alcohol	1 (0.6%)	4 (0.1%)	0.184 ^c^
Gestational age at cervical length measurement	Mean ± standard deviation (weeks)	21.5 ± 0.5	21.5 ± 0.6	0.592 ^a^
Type of pregnancy	Spontaneous	165 (95.4%)	4,041 (97.2%)	0.150 ^b^
	Medical assisted	8 (4.6%)	115 (2.8%)	
Gravidity	Primigravida	83 (46.5%)	1,953 (45.4%)	0.789 ^b^
Parity	Nulliparous	101 (56.4%)	2,417 (56.2%)	0.949 ^b^
Obstetric history	Preterm birth	17 (9.5%)	55 (1.3%)	< 0.001 ^c^
	Second trimester miscarriage	1 (0.6%)	4 (0.1%)	0.184 ^c^
	Recurrent pregnancy loss	2 (1.1%)	16 (0.4%)	0.160 ^c^
Maternal background	Conization	10 (5.6%)	31 (0.7%)	< 0.001 ^b^
	Mullerian anomalies	3 (1.7%)	11 (0.3%)	0.016 ^c^
	Chronic hypertension	9 (5.0%)	100 (2.3%)	0.021 ^b^
	Diabetes	3 (1.7%)	28 (0.7%)	0.125 ^c^
	Hypothyroidism	7 (3.9%)	227 (5.2%)	0.429 ^b^
Obstetrical complications	Malformations and cromossomopathies	6 (3.4%)	64 (1.5%)	0.059 ^c^
	Fetal growth restriction	9 (5.0%)	167 (3.9%)	0.439 ^b^
	Hypertensive syndromes	0	143 (3.3%)	0.040 ^c^
	Gestational diabetes	24 (1.4%)	405 (9.4%)	0.751 ^b^
	Short interpregnancy intervals	1 (0.6%)	4 (0.1%)	0.184 ^c^
	Surgical procedure during pregnancy	0 (0%)	7 (0.2%)	1.000 ^c^
	Urinary infections or asymptomatic bacteriuria	8 (4.5%)	135 (3.1%)	0.321 ^b^
	Others infections during pregnancy	10 (5.9%)	188 (4.4%)	0.438 ^b^
Treatment with progesterone or Arabin pessary		25 (13.9%)	116 (2.7%)	0.000 ^b^

Notes: ^a^ *t* -test; ^b^ Chi-squared test; ^c^ Fisher test.

**Table 5:** Cervical length description according to demographics

			Cervical length (mean ± standard deviation)	*p* -value ^a^
Maternal age	< 40 years old		33.8 ± 4.8	0.546
	≥ 40 years old		34.0 ± 5.0	
Schooling	≤ 12th grade		33.8 ± 4.8	0.568
	> 12th grade		33.9 ± 4.9	
Addictions	Smoking	No	33.9 ± 4.8	0.089
		Yes	33.5 ± 5.3	
	Drugs	No	33.8 ± 4.8	0.443
		Yes	35.5 ± 2.8	
	Alcohol	No	33.8 ± 4.8	0.170
		Yes	30.5 ± 3.1	
Type of pregnancy	Spontaneous		33.8 ± 4.8	0.664
	Medically-assisted		33.6 ± 6.4	
Gravidity	Primigravida		33.5 ± 4.8	0.000
	Multigravida		34.1 ± 4.9	
Parity	Nulliparous		33.4 ± 4.8	0.000
	Multiparous		34.3 ± 4.8	
Obstetric history	Preterm birth	No	33.9 ± 4.8	0.000
		Yes	31.8 ± 5.0	
	Second-trimester miscarriage	No	33.8 ± 4.8	0.001
		Yes	26.8 ± 8.4	
	Recurrent pregnancy loss	No	33.8 ± 4.8	0.412
		Yes	32.3 ± 7.9	
Maternal background	Conization	No	33.9 ± 4.8	0.000
		Yes	30.8 ± 5.6	
	Mullerian anomalies	No	33.8 ± 4.8	0.993
		Yes	33.9 ± 3.2	
	Chronic hypertension	No	33.8 ± 4.8	0.540
		Yes	34.1 ± 6.2	
	Diabetes	No	33.8 ± 4.8	0.120
		Yes	35.9 ± 4.6	
	Hypothyroidism	No	33.8 ± 4.9	0.659
		Yes	34.0 ± 4.5	
Obstetrical complications	Malformations and cromossomopathies	No	33.8 ± 4.8	0.821
		Yes	34.0 ± 4.9	
	Fetal growth restriction	No	33.9 ± 4.8	0.000
		Yes	32.4 ± 4.9	
	Hypertensive syndromes	No	33.8 ± 4.8	0.448
		Yes	33.5 ± 4.8	
	Gestational diabetes	No	33.8 ± 4.8	0.967
		Yes	33.8 ± 45.0	
	Short interpregnancy intervals	No	33.8 ± 4.8	0.871
		Yes	34.3 ± 2.4	
	Surgical procedure during pregnancy	No	33.8 ± 4.8	0.934
		Yes	34.0 ± 3.3	
	Urinary infections or asymptomatic bacteriuria	No	33.8 ± 4.8	0,112
		Yes	33.12 ± 4.8	
	Other infections during pregnancy	No	33.9 ± 4.9	0.880
		Yes	33.2 ± 4.6	
Treatment with progesterone or Arabin pessary		No	34.2 ± 4.3	0.000
		Yes	24.1 ± 7.2	

Note: ^a^ *t* -test.

The multivariate logistic regression analysis, incorporating maternal age ≥ 40 years old, history of PTB, and previous cervical surgery, evaluated the impact of the TUCL as a predictor of PTB ( Table 6 ).

**Table 6:** Multivariate logistic regression analysis to evaluate the impact of transvaginal ultrasound measurement of the cervical length as a predictor of preterm birth

Outcome	Odds ratio	95% confidence interval		*p* -value
Cervical length	0.925	0.90	0.95	0.000
Maternal age	2.265	1.32	3.90	0.003
Previous preterm birth	6.754	3.72	12.16	0.000
Cervical surgery	0.178	0.81	0.40	0.000
Constant	14.557	2.61	81.31	0.002

**Fig. 2:**
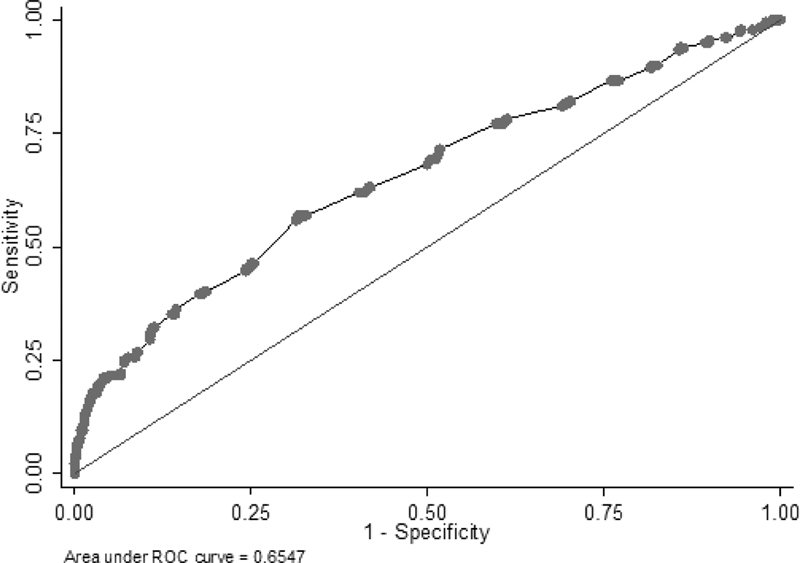
Graphic representations of sensitivity, specificity and area under the curve (AUC) of the multivariate model.

The estimated odds ratio (OR) for the effect of the TUCL on PTB, controlling for covariates, was of 0.92 (95%CI: 0.90–0.95; *p* < 0.001), which highlights the significant negative association between TUCL and PTB. The diagnostic ability of the multivariate model improved, showing an AUC of 0.65 ( Fig. 2 ).

As expected, in the univariate analysis, the treatment with progesterone/pessary was associated with shorter TUCL, and thus, also with PTB, when considering the total study cohort. Within the group of women with a diagnosis of short cervix, 68% (n = 93) underwent treatment, and 13% (n = 18) declined it. There was, however, no significant difference between the frequency of PTB among women who accepted or declined progesterone or the pessary (14% versus 22.2% of PTB respectively; *p* = 0.472); therefore, the treatment was not included in our multivariate model.

**Fig. 3:**
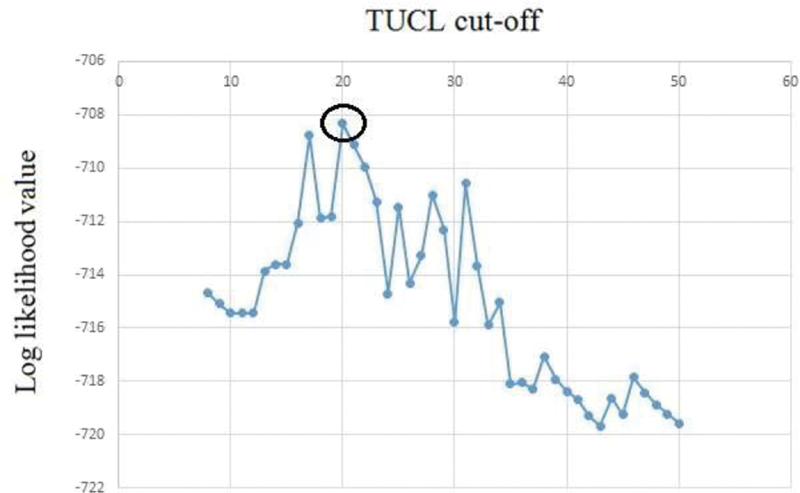
Plotting to determine the best TUCL cut-off based on multiple log likelihood of the logistic regression model (including maternal age ≥ 40 years, history of PTB, and previous cervical surgery), using cut-offs between 8 mm and 50 mm. The circle represents the cut-off that best discriminated the risk of experiencing PTB.

To select the optimal TUCL cut-off, we ran several multivariate logistic regression models using different cut-offs (from 8 mm and 50 mm) associated with other variables like maternal age ≥ 40 years, history of PTB, and previous cervical surgery. Plotting the log likelihood from these models against the TUCL showed that a cut-off of 20 mm (95%CI: 19.5–22 mm) best discriminated 2 TUCL subgroups with differential odds for PTB ( Fig. 3 ). Women with a short cervix, defined by TUCL ≤ 20 mm, had an OR of 12.2 (95%CI: 5.8–25.4; *p* < 0.001) compared with those with TUCL > 20 mm.

Considering maternal age ≥ 40 years, history of PTB and previous cervical surgery as the main risk factors for PTB, a stratified analysis was performed by separately evaluating women with at least 1 of these factors (n = 297) versus women who did not presented any of them (n = 4,184 [93.4%]) ( Table 7 ). The significant association of a short cervix (TUCL ≤ 20 mm) with PTB was maintained in both groups, with an OR of 16.2 (95%CI: 2.7–97.1; *p* < 0.001) for women with risk factors, and an OR of 9.8 (95%CI: 4.1–23.7; *p* < 0.001) for women without them. There was no effect modification between the groups ( *p* = 0.614, homogeneity test). Considering this stratification, the adjusted OR for women with short cervix (TUCL ≤ 20 mm) was of 11.4 (95%CI: 5.1–25.4; *p* < 0.001).

**Table 7:** Distribution of the pregnant women according to group probability of preterm birth using transvaginal ultrasound measurement of the cervical length and presence/absence of risk factors (maternal age ≥ 40 years, previous PTB and cervical surgery). Cross-classification of the 25-mm cut-off group (most used cut-off) versus the 20-mm cut-off group (our best cut-off) and frequency of PTB in each subgroup

		Group probability – TUCL: 20 mm				
		< 0.25	0.25-0.50	0.50-0.75	≥ 0.75	Total reclassified
Group probability – TUCL: 25 mm	< 0.25	4,456	1	0	0	0.02%
	PTB% (n)	3.8% (168)	0%	0%	0%	
	0.25-0.50	8	8	2	0	55.6%
	PTB% (n)	12.5% (1)	50.0% (4)	50.0% (1)	0%	
	0.50-0.75	0	0	1	4	80.0%
	PTB% (n)	0%	0%	100% (1)	75.0% (n3)	
	≥ 0.75	0	0	0	1	0%
	PTB% (n)	0%	0%	0%	0%	

Abbreviations: PTB, preterm birth; TUCL, transvaginal ultrasound measurement of the cervical length.

A simple comparison of the 20-mm and 25-mm cut-offs, based on AUCs of multivariate models, showed no statistical difference (20 mm: AUC = 0.59 [95%CI: 0.56–0.62] versus 25 mm: AUC = 0.60 [95%CI: 0.57–0.64]; *p* = 0.157). However, a better performance in the prediction of PTB of the 20-mm compared to the 25-mm cut-off was highlighted by comparing the distribution of women according to the prediction probabilities derived from the corresponding multivariate models. Globally, 15 (0.33%) women were reclassified to a different predicted-probability group when the multivariate model included the 20-mm instead of the 25-mm cut-off. As described in table 6 , out of the 4 women upgraded to a higher probability (from 0.50-0.75 in the 25-mm model to > 0.75 in the 20-mm model), 75% (n = 3) experienced a PTB. On the other hand, out of the 8 women reclassified to a lower predicted probability (from 0.25-0.50 in the 25-mm model to < 0.25 in the 20-mm model), only 1 (12.5%) had PTB.

## Discussion

Good practice of disease screening recommends that the condition be an important health problem and facilities for diagnosis and treatment be available, as already published in 1968 by the WHO. 28

Preterm birth fulfills the first prerequisite, as it represents a major obstetric complication. Our cohort presents 4% (179) of spontaneous PTB, and this prevalence is similar to that of other studies regarding screening and treatment. 27 29 30

A second assumption needed to implement a screening process is the existence of a test able to detect the high-risk population, and the TUCL satisfies this requirement. An inverse association between the TUCL and PTB, which was also observed in our study, has been widely documented. 16 21 26 27 29 30 31 32 The TUCL in our cohort showed a high specificity to predict PTB, but low sensitivity and a poor AUC, results similar to those of other studies. Iams et al. 27 reported that TUCL ≤ 25 mm had a sensitivity of 37% and a specificity of 92%, but more recent studies obtained even lower sensitivities, such as 2.4% in the study by van der Ven et al., 33 and 8.0% in the one by Esplin et al. 34

Our multivariate model showed an improvement in the AUC value, highlighting that a combined screening including maternal age > 40 years, history of PTB and previous cervical surgery should be considered for screening, instead of the TUCL alone. The Society for Maternal-Fetal Medicine Publications Committee, in their 2012 guidelines, concluded that the most effective approach was to initiate treatment in low-risk women with a TUCL ≤ 20 mm, or high-risk pregnant women with a TUCL ≤ 25 mm, supporting that other risk factors should be included in the screening algorithm. 35

Nowadays, there is no debate that second-trimester TUCL is the most powerful screening instrument available, but the best cut-off to separate normal from short cervixes is still controversial. 16 19 20 21 22 23 As most parameters in medicine, there is no biological TUCL cut-off, and defining “short” is not an easy task. Lower cut-offs present good specificity but low sensitivity, but higher values (like 29 mm) lead to an increase in the false-positive rate. 27 36 Most guidelines recommend a 25-mm cut-off, as it corresponds to the 10th percentile in the initial published trials. 7 15 16 27 36 37 38 39 40 However, more recent studies showed a lower prevalence of short cervices defined as TUCL ≤ 25 mm, averaging 2.5%. 14 33 34 41 42 Our results follow this new tendency, as the prevalence of TUCL ≤ 25 mm was of only 3% (134), and the 10th TUCL percentile in our cohort corresponded to 29.0 mm.

TUCL distribution can be influenced by many factors; therefore, the ideal cut-off can change in different populations. That said, we concluded that the best cut-off for our cohort was 20 mm ( Fig. 3 ). This value enabled us to improve the prediction of the risk of experiencing PTB mainly by reducing the false-positive rate (8 women were reclassified as low probability, and only 1 (15.5%) of them experienced a PTB).

In parallel to studies on the efficacy of TUCL screening, cost-analysis studies were also conducted, which concluded that TUCL screening is cost-effective even if we assume a low incidence of short cervical length and a modest impact of the treatment with progesterone. 25 31 43 44 45

The Federation of Gynecology and Obstetrics Working Group on Best Practices in Maternal Fetal Medicine recommended universal transvaginal cervical length screening and vaginal progesterone when TUCL< 25 mm. 46 Subsequently, studies 41 42 47 using this recommendation showed a reduction in the PTB rate when universal screening was applied. Son et al., 42 for example, obtained a 20% reduction in the rate of PTBs after implementing TUCL screening, even with a very low prevalence short cervixes (TUCL ≤ 25 mm: 0.89%). The negative impact of PTB is so huge that every approach able to reduce it has a positive impact and should be considered.

## Conclusion

Preterm birth represents a major health problem, and strategies to prevent are important. The present study showed an inverse association between TUCL and PTB, and emphasized that other factors like maternal age, history PTB and previous cervical surgery can improve the screening algorithm. The value that defines a short cervix can differ in each population, and, for our cohort, the best cut-off was 20 mm. Even though TUCL has a low diagnostic performance, it is the best screening method available to predict PTB, and TUCL screening has been shown to reduce the PTB rate.

## References

[1] Glover A V Manuck T A Screening for spontaneous preterm birth and resultant therapies to reduce neonatal morbidity and mortality: A review Semin Fetal Neonatal Med 2018 23 02 126 132 10.1016/j.siny.2017.11.007 29229486 PMC6381594

[2] Purisch S E Gyamfi-Bannerman C Epidemiology of preterm birth Semin Perinatol 2017 41 07 387 391 10.1053/j.semperi.2017.07.009 28865982

[3] Vogel J P Chawanpaiboon S Moller A B Watananirun K Bonet M Lumbiganon P The global epidemiology of preterm birth Best Pract Res Clin Obstet Gynaecol 2018 52 3 12 10.1016/j.bpobgyn.2018.04.003 29779863

[4] Euro-Peristat Preterm Study Group Zeitlin J Szamotulska K Drewniak N Preterm birth time trends in Europe: a study of 19 countries BJOG 2013 120 11 1356 1365 10.1111/1471-0528.12281 23700966 PMC4285908

[5] Frey H A Klebanoff M A The epidemiology, etiology, and costs of preterm birth Semin Fetal Neonatal Med 2016 21 02 68 73 10.1016/j.siny.2015.12.011 26794420

[6] Liu L Oza S Hogan D Global, regional, and national causes of under-5 mortality in 2000-15: an updated systematic analysis with implications for the Sustainable Development Goals Lancet 2016 388(10063):3027 3035 10.1016/S0140-6736(16)31593-8 27839855 PMC5161777

[7] Committee on Practice Bulletins—Obstetrics, The American College of Obstetricians and Gynecologists Practice bulletin no. 130: prediction and prevention of preterm birth Obstet Gynecol 2012 120 04 964 973 10.1097/AOG.0b013e3182723b1b 22996126

[8] Dodd J M Jones L Flenady V Cincotta R Crowther C A Prenatal administration of progesterone for preventing preterm birth in women considered to be at risk of preterm birth Cochrane Database Syst Rev 2013 07 CD004947 10.1002/14651858.CD004947 23903965 PMC11035916

[9] Born Too Soon Preterm Birth Action Group Howson C P Kinney M V McDougall L Lawn J E Born too soon: preterm birth matters Reprod Health 2013 10 01 S1 10.1186/1742-4755-10-S1-S1 24625113 PMC3828581

[10] neonatal health research priority setting group Yoshida S Martines J Lawn J E Setting research priorities to improve global newborn health and prevent stillbirths by 2025 J Glob Health 2016 6 01 10508 10.7189/jogh.06.010508 PMC4576458 26401272

[11] Loureiro T Cunha M Montenegro N [Sonographic measurement of cervical length and prediction of spontaneous preterm delivery: how useful is it?] Acta Med Port 2006 19 05 395 404Portuguese 17376326

[12] Barde D MPS Attal D MP Transvaginal sonographic cervical length measurement as predictor of preterm delivery Int J Med Sci Clin Invent 2017 4 07 3129 3132 10.18535/ijmsci/v4i7.17

[13] Krupa F G Faltin D Cecatti J G Surita F GC Souza J P Predictors of preterm birth Int J Gynaecol Obstet 2006 94 01 5 11 10.1016/j.ijgo.2006.03.022 16730012

[14] Temming L A Durst J K Tuuli M G Universal cervical length screening: implementation and outcomes Am J Obstet Gynecol 2016 214 04 5230 5.23E10 10.1016/j.ajog.2016.02.002 PMC4810783 26874299

[15] Society for Maternal-Fetal Medicine (SMFM). Electronic address: pubs@smfm.org McIntosh J Feltovich H Berghella V Manuck T The role of routine cervical length screening in selected high- and low-risk women for preterm birth prevention Am J Obstet Gynecol 2016 215 03 B2 B7 10.1016/j.ajog.2016.04.027 27133011

[16] Andersen H F Nugent C E Wanty S D Hayashi R H Prediction of risk for preterm delivery by ultrasonographic measurement of cervical length Am J Obstet Gynecol 1990 163 03 859 867 10.1016/0002-9378(90)91084-p 2206073

[17] Leung T N Pang M W Leung T Y Poon C F Wong S M Lau T K Cervical length at 18-22 weeks of gestation for prediction of spontaneous preterm delivery in Hong Kong Chinese women Ultrasound Obstet Gynecol 2005 26 07 713 717 10.1002/uog.2617 16308894

[18] Burger M Weber-Rössler T Willmann M Measurement of the pregnant cervix by transvaginal sonography: an interobserver study and new standards to improve the interobserver variability Ultrasound Obstet Gynecol 1997 9 03 188 193 10.1046/j.1469-0705.1997.09030188.x 9165682

[19] van Os M A Kleinrouweler C E Schuit E Influence of cut-off value on prevalence of short cervical length Ultrasound Obstet Gynecol 2017 49 03 330 336 10.1002/uog.15967 27194622

[20] Wulff C B Rode L Rosthøj S Hoseth E Petersen O B Tabor A Transvaginal sonographic cervical length in first and second trimesters in a low-risk population: a prospective study Ultrasound Obstet Gynecol 2018 51 05 604 613 10.1002/uog.17556 28639717

[21] Markham K B Iams J D Measuring the cervical length Clin Obstet Gynecol 2016 59 02 252 263 10.1097/GRF.0000000000000204 27042799

[22] Verma S Meena B S Pooja P Sehra R N A study of cervical length measured ultrasonographically in prediction of preterm delivery J Obstet Gynaecol 2017 3 04 38 43

[23] Orzechowski K M Nicholas S S Baxter J K Weiner S Berghella V Implementation of a universal cervical length screening program for the prevention of preterm birth Am J Perinatol 2014 31 12 1057 1062 10.1055/s-0034-1371710 24705970

[24] Berghella V Baxter J K Hendrix N W Cervical assessment by ultrasound for preventing preterm delivery Obstet Gynecol 2009 114 05 1140 1141 10.1097/AOG.0b013e3181bdca73 20168118

[25] Jain S Kilgore M Edwards R K Owen J Revisiting the cost-effectiveness of universal cervical length screening: importance of progesterone efficacy Am J Obstet Gynecol 2016 215 01 1010 1.01E9 10.1016/j.ajog.2016.01.165 26821336

[26] Barros-Silva J Pedrosa A C Matias A Sonographic measurement of cervical length as a predictor of preterm delivery: a systematic review J Perinat Med 2014 42 03 281 293 10.1515/jpm-2013-0115 24169309

[27] National Institute of Child Health and Human Development Maternal Fetal Medicine Unit Network Iams J D Goldenberg R L Meis P J The length of the cervix and the risk of spontaneous premature delivery N Engl J Med 1996 334 09 567 572 10.1056/NEJM199602293340904 8569824

[28] Wilson J MG Jungner G Principles and practice of screening for disease Geneva World Health Organization 1968

[29] Kuusela P Jacobsson B Söderlund M Transvaginal sonographic evaluation of cervical length in the second trimester of asymptomatic singleton pregnancies, and the risk of preterm delivery Acta Obstet Gynecol Scand 2015 94 06 598 607 10.1111/aogs.12622 25732204

[30] Souka A P Papastefanou I Pilalis A Kassanos D Papadopoulos G Implementation of universal screening for preterm delivery by mid-trimester cervical-length measurement Ultrasound Obstet Gynecol 2019 53 03 396 401 10.1002/uog.19050 29536576

[31] Pedretti M K Kazemier B M Dickinson J E Mol B W Implementing universal cervical length screening in asymptomatic women with singleton pregnancies: challenges and opportunities Aust N Z J Obstet Gynaecol 2017 57 02 221 227 10.1111/ajo.12586 28295170

[32] Conde-Agudelo A Romero R Vaginal progesterone to prevent preterm birth in pregnant women with a sonographic short cervix: clinical and public health implications Am J Obstet Gynecol 2016 214 02 235 242 10.1016/j.ajog.2015.09.102 26450404 PMC5703061

[33] van der Ven J van Os M A Kazemier B M The capacity of mid-pregnancy cervical length to predict preterm birth in low-risk women: a national cohort study Acta Obstet Gynecol Scand 2015 94 11 1223 1234 10.1111/aogs.12721 26234711

[34] nuMoM2b Network Esplin M S Elovitz M A Iams J D Predictive accuracy of serial transvaginal cervical lengths and quantitative vaginal fetal fibronectin levels for spontaneous preterm birth among nulliparous women JAMA 2017 317 10 1047 1056 10.1001/jama.2017.1373 28291893 PMC5828036

[35] Society for Maternal-Fetal Medicine Publications Committee, with assistance of Vincenzo Berghella Progesterone and preterm birth prevention: translating clinical trials data into clinical practice Am J Obstet Gynecol 2012 206 05 376 386 10.1016/j.ajog.2012.03.010 22542113

[36] Taipale P Hiilesmaa V Sonographic measurement of uterine cervix at 18-22 weeks' gestation and the risk of preterm delivery Obstet Gynecol 1998 92 06 902 907 10.1016/s0029-7844(98)00346-9 9840546

[37] Hassan S S Romero R Berry S M Patients with an ultrasonographic cervical length < or =15 mm have nearly a 50% risk of early spontaneous preterm delivery Am J Obstet Gynecol 2000 182 06 1458 1467 10.1067/mob.2000.106851 10871466

[38] Romero R Conde-Agudelo A Da Fonseca E Vaginal progesterone for preventing preterm birth and adverse perinatal outcomes in singleton gestations with a short cervix: a meta-analysis of individual patient data Am J Obstet Gynecol 2018 218 02 161 180 10.1016/j.ajog.2017.11.576 29157866 PMC5987201

[39] Fetal Medicine Foundation Second Trimester Screening Group Fonseca E B Celik E Parra M Singh M Nicolaides K H Progesterone and the risk of preterm birth among women with a short cervix N Engl J Med 2007 357 05 462 469 10.1056/NEJMoa067815 17671254

[40] Berghella V Palacio M Ness A Alfirevic Z Nicolaides K H Saccone G Cervical length screening for prevention of preterm birth in singleton pregnancy with threatened preterm labor: systematic review and meta-analysis of randomized controlled trials using individual patient-level data Ultrasound Obstet Gynecol 2017 49 03 322 329 10.1002/uog.17388 27997053

[41] Orzechowski K M Boelig R C Baxter J K Berghella V A universal transvaginal cervical length screening program for preterm birth prevention Obstet Gynecol 2014 124 03 520 525 10.1097/AOG.0000000000000428 25162252

[42] Son M Grobman W A Ayala N K Miller E S A universal mid-trimester transvaginal cervical length screening program and its associated reduced preterm birth rate Am J Obstet Gynecol 2016 214 03 3650 3.65E7 10.1016/j.ajog.2015.12.020 26928150

[43] Crosby D A Miletin J Semberova J Daly S Is routine transvaginal cervical length measurement cost-effective in a population where the risk of spontaneous preterm birth is low? Acta Obstet Gynecol Scand 2016 95 12 1391 1395 10.1111/aogs.13021 27623283

[44] Einerson B D Grobman W A Miller E S Cost-effectiveness of risk-based screening for cervical length to prevent preterm birth Am J Obstet Gynecol 2016 215 01 1000 1.0E9 10.1016/j.ajog.2016.01.192 26880732

[45] Werner E F Hamel M S Orzechowski K Berghella V Thung S F Cost-effectiveness of transvaginal ultrasound cervical length screening in singletons without a prior preterm birth: an update Am J Obstet Gynecol 2015 213 04 5540 5.54E8 10.1016/j.ajog.2015.06.020 26071914

[46] Figo Working Group On Best Practice In Maternal-Fetal Medicine International Federation of Gynecology and Obstetrics Best practice in maternal-fetal medicine Int J Gynaecol Obstet 2015 128 01 80 82 10.1016/j.ijgo.2014.10.011 25481030

[47] Newnham J P White S W Meharry S Reducing preterm birth by a statewide multifaceted program: an implementation study Am J Obstet Gynecol 2017 216 05 434 442 10.1016/j.ajog.2016.11.1037 27890647

